# Comparative analysis of differentially expressed miRNAs related to uterine involution in the ovine ovary and uterus

**DOI:** 10.5194/aab-64-167-2021

**Published:** 2021-05-12

**Authors:** Heng Yang, Lin Fu, Qifeng Luo, Licai Li, Fangling Zheng, Jiayu Wen, Xingxiu Luo, Chenjing Li, Zongsheng Zhao, Huihao Xu, Gaofu Wang

**Affiliations:** 1 College of Veterinary Medicine, Southwest University, Rongchang 402460, Chongqing, China; 2 Immunology Research Center, Medical Research Institute, Southwest University, Rongchang 402460, Chongqing, China; 3 Chongqing academy of animal sciences, Rongchang 402460, Chongqing, China; 4 College of Animal Science and Technology, Shihezi University, Shihezi 832000, Xinjiang, China

## Abstract

To examine the possible miRNA molecular regulatory mechanisms during maternal uterine involution after delivery, we selected ovary and uterus tissues that are structurally connected as experimental materials. We employed Illumina HiSeq sequencing to screen and analyze the quantity and characteristics of miRNA in postpartum ewes in the methylergometrine-treated group and physiological saline control group. Results showed that 16 miRNAs were identified in the ovary libraries, including 4 known miRNAs and 12 novel miRNAs. In the uterus libraries, 54 miRNAs were identified, which included 5 known miRNAs and 49 novel miRNAs. At the same time, target gene prediction, GO annotation, and KEGG signaling pathway enrichment analysis were employed. We found that maternal uterine involution after delivery may involve two miRNA-target gene pairs, i.e., miRNA-200a-*ZEB1* and *YAP1*. The YAP1/Hippo signaling pathway is used to construct an ovary–uterine axial regulatory mechanism to regulate the restoration of postpartum maternal uterine morphology and function. In view of this, the identification of miRNAs with significant differences in this study fills a gap in research on miRNAs associated with regulation of postpartum uterine recovery in ewes and provided an important reference for comprehensive understanding and in-depth research on the regulatory molecular network mechanism for postpartum uterine involution in small ruminants.

## 1 Introduction

Postpartum maternal reproductive function restoration usually involves two aspects, including the recovery of ovarian function and completed uterine involution. Among the uterus and ovaries, the uterus is the reproductive organ that experiences the greatest change in morphology and function as it showed endometrial regeneration and reconstruction of uterine function. The recovery of maternal uterus after delivery to a non-pregnant state is known as uterine involution. This plays a vital role in ensuring normal reproductive function and cyclical estrus in postpartum female animals (Sheldon and Dobson, 2004; Mahdi and Khallili, 2008).

The uterus is a dynamic and complex organ that interacts with many deep tissues, ovaries, cerebrum, and other organs. In many of the aforementioned connections, it is evident that the uterus and ovaries are dependent and interact with each other in morphology and regulation of endocrine secretion. Structurally, the ovaries are located at the posterolateral side at the end of the uterus and are connected to the uterus through Fallopian tubes. There are many nerve plexi and blood vessels between the uterus and ovaries. At the uterus, the internal iliac artery supplies the uterus and is anastomosed with the ovarian arteries (branch of the aorta) and is connected to the inferior and superior vaginal blood vessels. At the same time, the lymph nodes of the uterine body are extended to the pelvic cavity on one side and the para-aortic lymph nodes on the other side (Ercoli et al., 2010; Lanciego et al., 2012; Abe et al., 2014; Hamadeh et al., 2018). At the endocrine secretion level, the ovaries synthesize and secrete steroid hormones such as E${}_{2}$ and P${}_{4}$. These two hormones are transported to uterine tissues via the utero-ovarian plexus (UOP) and play important roles in the proliferation, differentiation, and functional changes of the endometrium and myometrium. A study showed that E${}_{2}$ not only stimulates myometrial activity, promotes uterine muscle excitation, and accelerates uterine muscle contraction and action potential frequencies of individual fibers (Batra, 1980), but also promotes intrauterine PGF${}_{2\alpha }$ secretion, OTR synthesis and collagenase activity, and cervix maturation. Similarly, P${}_{4}$ secreted by the corpus luteum not only inhibits cervix maturation and myometrial excitation but also simultaneously decreases myometrial spontaneous electrical activity and OXT sensitivity, thereby affecting the physiological functions of the uterus (Hafez and Hafez, 2000; Rodriguez Blanquet, 2003). Conversely, PGF${}_{2\alpha }$ and/or PGE${}_{2}$ synthesized and released by uterine tissues are secreted into uterine veins and are locally transported into ovarian tissues through the unique vascular structure of the UOP to regulate corpus luteum maintenance and degradation. Therefore, changes in the structures and functions of these tissues created preconditions for ensuring the cyclicity and continuity in breeding of female animals in the husbandry industry.

In recent years, a large volume of studies found that smooth muscle cells are rich in many types of stable miRNAs, and these miRNAs play important roles in the proliferation, hypertrophy, and differentiation of smooth muscle cells (Carletti and Christenson, 2009; Li, 2014; Nothnick, 2015). This suggests that a large number of miRNAs may participate in postpartum maternal uterine involution, particularly the recovery of uterine smooth muscle contraction function, and these miRNAs may play specific regulatory roles during this process. In view of this, we employed miRNA HiSeq deep sequencing to construct miRNA libraries of the ovary and uterus of postpartum ewes from treatment group (intramuscular injection of ergometrine) and control group (intramuscular injection of physiological saline) to screen miRNAs in the ovary–uterus axis that participate in postpartum maternal uterine involution. GO annotation and KEGG signaling pathway analysis were used to predict target genes and the networks regulated by these genes. The results provide an important reference for comprehensive understanding and examining of the molecular network regulatory mechanisms in postpartum maternal uterine involution.

## 2 Materials and methods

### 2.1 Animals and experiment design

Forty healthy adult Kazakh ewes that had similar ages (3–4 years), weighing 45–50 kg, free from uterine diseases, and with normal reproductive function were selected. The ewes were housed in the Shihezi University experimental station under the same housing environment. Methylergometrine and physiological saline were injected intramuscularly, and postpartum uterine involution model construction was completed to ensure significant phenotypic changes in uterine involution in postpartum ewe populations. There were 15 ewes in the treatment group, and 0.2 mg ergometrine was injected into the medial thigh in the rear limb at 1 d after delivery. There were 15 ewes in the control group, and an equal volume of physiological saline for medical use was injected in the same site 1 d after delivery. Following that, dynamic changes in the uterus were monitored every day. In addition, early weaning was carried out 7 d after delivery in which the ewes and lambs in the aforementioned groups were separated (lambs were transferred to another pen for milk replacer feeding by dedicated staff).

### 2.2 Sample collection

The Tringa Vet portable B-mode ultrasound device was used to monitor uterine involution in postpartum ewes. Uterine involution was considered to have ended when the uterine cavity was completely sealed, no residual liquid was present in the uterine cavity, and uterus diameter was less than 2 cm. Following that, three postpartum ewes that met the aforementioned criteria were selected from each group and recorded as the ergometrine-treated group with fast uterine involution (UF) and physiological saline-treated group with slow uterine involution (US). After ewes were sacrificed, the ovary and uterus were collected, numbered, and placed in liquid nitrogen for storage before sampling.

### 2.3 Total RNA extraction and quality testing

After the aforementioned samples were collected, chloroform, isopropanol, and 70 % ethanol were used to extract total RNA from the samples. Following that, total RNA from the UF and US group samples was sent to Honortech Co. Ltd. on dry ice, and the Agilent 2100 Bioanalyzer, Kaiao K5500 microspectrophotometer, and Agilent RNA 6000 Nano Kit were used to measure RNA integrity and purity. Qualified samples were used to construct ovary and uterus miRNA libraries.

### 2.4 MiRNA library construction and testing

The aforementioned postpartum ovine ovary and uterus miRNA libraries in the UF and US groups with constructed uterine involution were marked as fast uterine involution–ovary (UFO) vs. slow uterine involution–ovary (USO), fast uterine involution–uterus (UFU) vs. slow uterine involution–uterus (USU). Following that, the Agilent 2100 Bioanalyzer and ABI StepOnePlus Real-Time PCR System were used for quality and yield measurements of the constructed libraries. Lastly, the Illumina HiSeq 2500 platform was used for high-throughput sequencing of qualified sequencing libraries using the SE50 sequencing strategy.

### 2.5 Bioinformatics analysis

The different sequences obtained after sequencing were first filtered to obtain reliable target sequences, and the quality and lengths of these sequences and inter-sample common sequences were tallied. Following that, target sequences were classified and annotated to obtain the various component and expression level information in each sample. Annotated small RNA fragments were used for the prediction of novel miRNAs. At the same time, log2 ratio and scatterplot were used to analyze and screen differentially expressed miRNAs between groups. Lastly, the selected differentially expressed known miRNAs and novel miRNAs were used for cluster analysis, target gene prediction, and GO functional annotation and KEGG pathway annotation of target genes.

### 2.6 Validation of quantitative real-time polymerase chain reaction (qRT-PCR)

Twelve miRNAs, each with six miRNAs in the ovary and uterus libraries, were randomly selected to verify the reliability of the sequencing results. miRNAs and mRNAs were reverse-transcribed to cDNA with the miRcute miRNA First-strand cDNA Kit (TIANGEN, Beijing, China). qRT-PCR was then performed using the LightCycler96 qRT-PCR System (Stratagene, USA) and miRcute miRNA Premix SYBR (TIANGEN), following the manufacturer's instructions. All reactions were performed in triplicate. U6 RNA was chosen as an endogenous internal control, and the relative expression levels were calculated based on the 2${}^{-\Delta \Delta \mathrm{Ct}}$ method. The miRNA-specific primers are listed in Table S1A and B in the Supplement.

### 2.7 Differential expression of miRNA-target gene pairs in ovary and uterus

Based on the joint analysis of all miRNA libraries, the proposed candidate miRNAs and potential target genes were finally selected, and total RNA was extracted from different tissues of the same as the UF vs. US group and batch for real-time fluorescence detection of miRNAs and mRNA. These potential target gene primers are listed in Table S1C. All experiments were repeated three times with three replicates for each sample.

## 3 Results and analysis

### 3.1 Measurement of morphological changes during uterine involution

**Figure 1:**
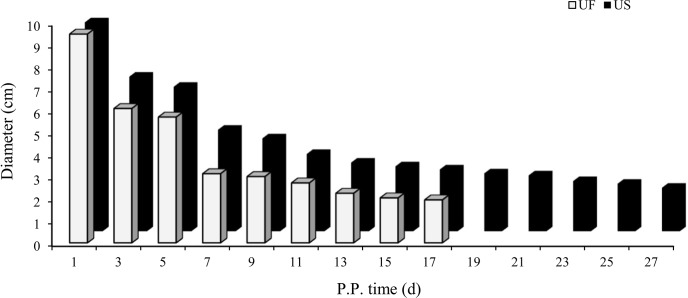
Trends for maximum cross-sectional diameter of the postpartum uterine horn between UF and US group.

B-mode ultrasound is an effective and reliable method to monitor postpartum uterine involution status in sheep. Two days after ewe delivery, the uterine horns were large in volume and contain more lochia. A linear array probe B-mode ultrasound was used for transrectal examination of postpartum uterine involution changes but cannot completely observe the status of the entire uterus. From day 3 after delivery onwards, the uterine horns of postpartum ewes showed significant contraction. However, after the cervix and uterine body have completely closed, the uterine cavity in the uterine horn was still partially opened. This shows that completion of uterine horn recovery can be used as a marker for uterine recovery. Based on the aforementioned uterine involution determination criteria, we used B-mode ultrasound to track and monitor changes in the largest cross section during uterine horn recovery in the postpartum ewes (Fig. 1). Results showed that the uterine involution duration in the UF group (17 d) was significantly shorter than the US group (27 d). This difference in uterine involution duration between postpartum ewes from the two groups was significantly different, proving that construction of animal models for different duration of uterine involution was successful and can be used for subsequent experiments.

### 3.2 Sequence quality preprocessing

To ensure accuracy in experimental data and improve the quality of data obtained, data preprocessing was carried out based on annotation information to finally obtain final clean reads for the UF and US group samples. Results showed that 41 262 294, 42 539 226, 42 355 026, and 40 960 450 raw reads were obtained from the UFO, USO, UFU, and USU libraries, respectively. Following that, adapters, contaminated sequences, and low-quality reads were removed to obtain clean data, and Rfam and miRbase database annotation were used for data preprocessing to obtain 40 321 542 and 40 702 382 clean reads from the UFO and USO libraries respectively, which accounted for 97.72 % and 95.68 % of raw reads, respectively (Table 1). Similarly, 40 978 134 and 39 652 778 clean reads were obtained from the UFU and USU libraries, respectively, which accounted for 96.75 % and 96.81 % of total reads, respectively (Table 1). This shows that the quality of miRNA libraries constructed was good and can be used for subsequent analysis.

**Table 1:** Percentage and distribution of sequencing results in four libraries.

Reads type	Raw reads	Clean reads	
		Number	(clean reads/
			raw reads) %
UFO	41 262 294	40 321 542	97.72 %
USO	42 539 226	40 702 382	95.68 %
UFU	42 355 026	40 978 134	96.75 %
USU	40 960 450	39 652 778	96.81 %

### 3.3 Small RNA sequence classification and annotation

**Figure 2:**
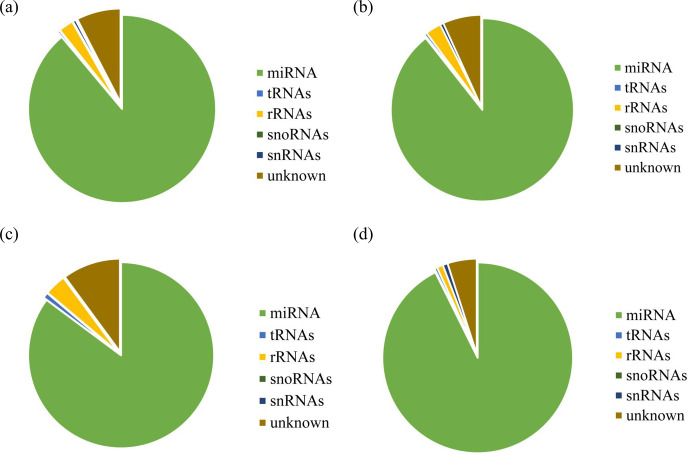
Statistics of the distribution characteristics of non-coding small RNAs between UF and US group. **(a)** UFO, **(b)** USO, **(c)** UFU, and **(d)** USU.

After removing contamination, all clean reads that were 18 nt and above in length were used for genome localization, classification, and annotation. First, small RNA sequences were aligned with non-coding small RNA and RNA repeat sequences, introns, and exons in Rfam database 11.0 and NCBI GenBank database. Results are shown in Fig. 2a, b, c, and d. Alignment results showed that most of the small RNAs in the UFO (Fig. 2a), USO (Fig. 2b), UFU (Fig. 2c), and USU (Fig. 2d) databases were miRNAs. Following that, known and unknown miRNAs in the two databases were used as data sources for subsequent analysis.

### 3.4 Analysis of differentially expressed miRNAs between UF and US group

Compared with the US group, 16 significant differentially expressed miRNAs were found in the UFO libraries, of which 4 and 12 were known and unknown miRNAs, respectively. Among these miRNAs, the four known miRNAs were upregulated (Table 2). At the same time, 54 significant differentially expressed miRNAs were found in the UFU libraries, of which 5 and 49 were known and unknown miRNAs, respectively. Among these five known miRNAs, two were upregulated, and three were downregulated. Table 2 shows some known differentially expressed miRNAs. It should be worth noting that we found one common differentially expressed miRNA in the ovary and uterus libraries, which was oar-miR-200a. In addition, Table 3 shows the position and mature sequences of the 12 most significant differentially expressed unknown miRNAs from the UF and US libraries in the genome.

**Table 2:** The significantly differential expression of known miRNA.

Samples	miRNAs	Log FC (UF/US)	P value	Significance label
Ovary	oar-miR-200a	3.852934599	$1.54\times 10^{-7}$	${}^{**}$
	oar-miR-200c	3.991774481	$1.68\times 10^{-7}$	${}^{**}$
	oar-miR-200b	3.405200876	$3.85\times 10^{-6}$	${}^{**}$
	oar-miR-150	1.490556611	0.027201507	${}^{*}$
Uterus	oar-miR-379-5p	1.931280281	0.005281951	${}^{**}$
	oar-miR-665-3p	1.851498825	0.015859258	${}^{**}$
	oar-miR-200a	-1.52039599	0.023035785	${}^{*}$
	oar-miR-133	-1.515683829	0.024476599	${}^{*}$
	oar-miR-99a	-1.306324483	0.04920584	${}^{*}$

**Table 3:** Relatively higher abundance 12 novel miRNA position on the genome and its sequence in UF and US libraries.

Groups	miRNA	Mature sequence (5${}^{\prime }$–3${}^{\prime }$)	Chromosome localization	Free energy
				kcal/mol
UFO vs. USO	oar-novel-miR-298-5p	UGGCAGUGUAUUGUUAGCUGGU	Chr 16:24003121-24003183	-24.8
	oar-novel-miR-977-5p	AGGCAGUGUAUUGUUAGCUGGCU	Chr 16:24003000-24003062	-23.3
	oar-novel-miR-296-5p	UCCUUCAUUCCACCGGAGUCUG	Chr 12:72034197-72034258	-25.7
	oar-novel-miR-481-5p	UCCUUCAUUCCACCGGAGUCUGU	Chr 12:72034198-72034258	-25.7
	oar-novel-miR-457-5p	AGGCAGUGCAUCUCUAGCUGGCU	Chr 16:24001790-24001853	-28.5
	oar-novel-miR-158-5p	CCCGGUACUGAGCUGACCCGAG	Chr 26:35066722-35066785	-36.7
UFU vs. USU	oar-novel-miR-555-5p	UGGACGGAGAACUGAUAAGGGU	Chr 18:24440152-24440240	-26.1
	oar-novel-miR-1185-3p	UGGAAUGUAAAGAAGUAUGUAU	Chr 23:34763997-34764058	-20.9
	oar-novel-miR-719-5p	CCGCGGCGGGGGCGGUCC	Chr 3:221307563-221307632	-38.4
	oar-novel-miR-378-3p	UAACUGUGGCGCAUGGGCUUCA	Chr 2:11892272-11892346	-27.5
	oar-novel-miR-1119-5p	GUGGACUUCCCUGGUAGCUCAGC	Chr 2:157399570-157399661	-40.2
	oar-novel-miR-1210-3p	UUAUUGCUUAAGAAUACGCGUAGU	Chr 1:74528676-74528737	-24.6

### 3.5 Real-time fluorescence quantitative PCR validation of the libraries

**Figure 3:**
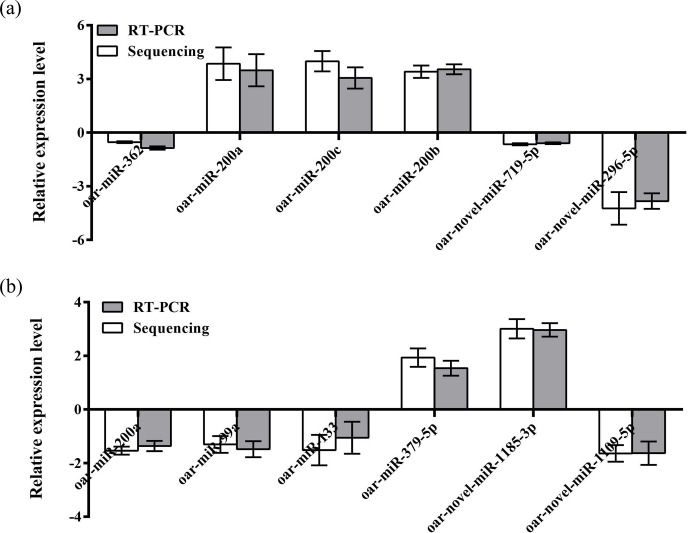
Validation of the Solexa sequencing results in the ovarian and uterine libraries. Data are mean ± SEM. Panels **(a)** and **(b)** show ovary and uterus libraries, respectively.

To further validate the reliability of the four miRNA libraries, we randomly selected six differentially expressed miRNAs from the ovary and uterus libraries, including four known miRNAs and two unknown miRNAs for RT-PCR validation. The 2${}^{-\Delta \Delta \mathrm{Ct}}$ method was used to calculate the relative expression level of the aforementioned miRNAs (Fig. 3a and b). Results showed that the expression level differences and library sequencing high-throughput differences of the six miRNAs were consistent, indicating that the ovary and uterus library data obtained are trustworthy and reliable.

### 3.6 Prediction and analysis of miRNA target genes

**Figure 4:**
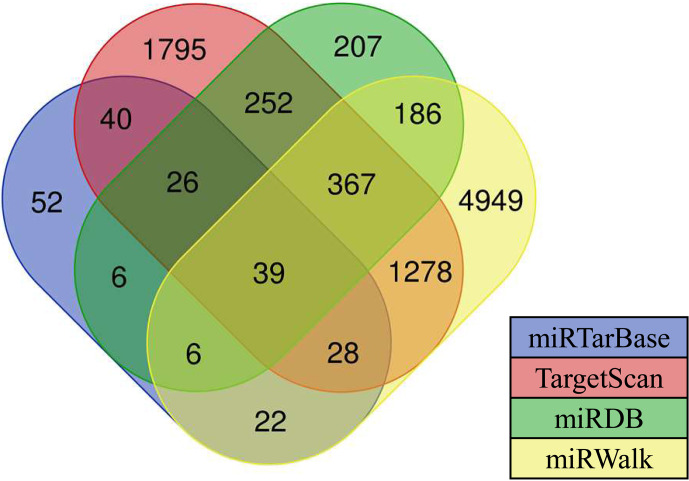
Various types of software for predicting the target genes of miRNAs. The numbers in the intersection of the four circles refer to the candidate target genes of miRNAs.

Currently, there are a few software programs used for the prediction of miRNA targets. In this study, four software programs (such as TargetScan, miRDB, miRWalk, and miRTarBase) were used to predict miRNA target genes, and the intersecting target genes were finally selected as target genes for candidate miRNAs. Results showed that there are 39 target genes of differentially expressed oar-miR-200a that are common to the ovary and uterus libraries (Fig. 4). Preliminary screening and analysis were used for GO annotation and KEGG pathway enrichment analysis of the aforementioned target genes.

### 3.7 GO enrichment analysis and KEGG pathway analysis of target genes

**Figure 5:**
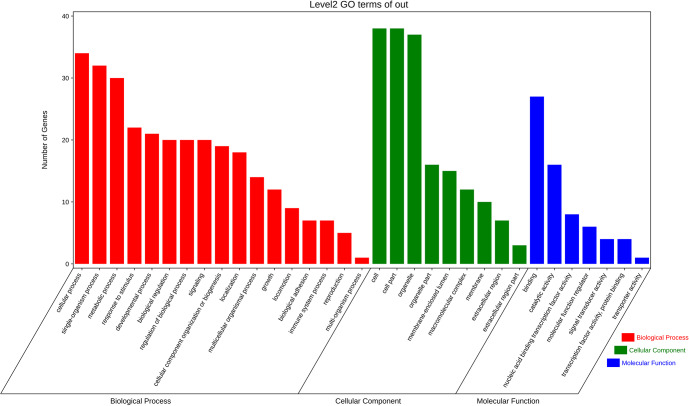
All the target genes were analyzed using taxonomic annotation and enrichment analysis in terms of molecular function, biological process, and cell composition.

**Figure 6:**
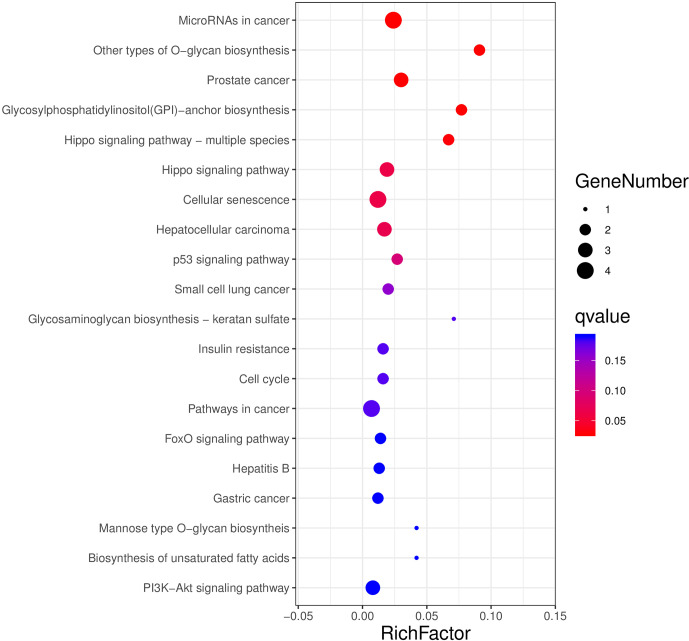
All the target genes were conducted using KEGG signaling pathway enrichment analysis. P value < 0.05 was considered statistically significant.

The aforementioned 39 target gene sets were used for molecular function, biological process, and cellular component annotation and enrichment analysis. Results showed that GO enrichment of target genes in this study are involved in the regulation of many biological processes, such as reproduction, multi-organism process, biological adhesion, and biological regulation (Fig. 5). At the same time, KEGG signaling pathway enrichment analysis of the above target genes found that a large number of candidate genes are enriched in microRNAs in cancer, glycosylphosphatidylinositol (GPI)-anchor biosynthesis, Hippo signaling pathway, and Hippo signaling pathway-multiple species (Fig. 6).

### 3.8 Tissue expression spectrum

**Figure 7:**
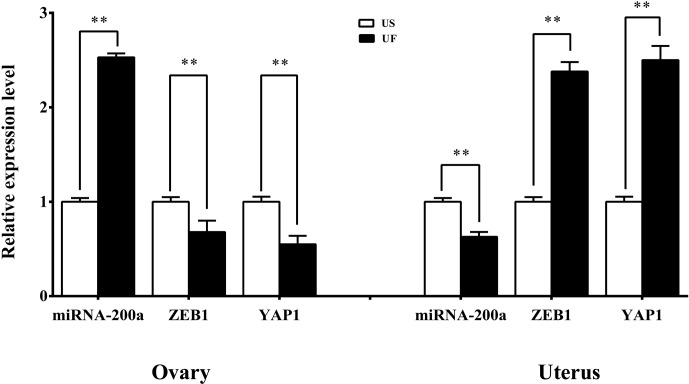
Expression levels of the candidate miRNAs and their target genes in the ovarian and uterine samples. All the samples are consistent with the library samples.

GO and KEGG enrichment analyses based on miRNA target prediction and target genes found that one miRNA-target gene pair was involved in microRNAs in cancer, which was miR-200a-*ZEB1*. At the same time, another miRNA-target gene pair is involved in the Hippo signaling pathway, i.e., miR-200a-*YAP1*. Based on this, we used the same treatment group tissue samples for qRT-PCR for further validation of the reliability of the aforementioned screening results (Fig. 7). Results showed that significant differential expression of miRNA-200a and its potential target genes (*ZEB1* and *YAP1*) are present in ovary and uterus tissues, and the expression trends of the two are negatively correlated. This shows that typical miRNA-target gene negative regulatory relationships may be present between them. This provided a direction and basis for subsequent mining of functional regulatory mechanisms.

## 4 Discussion

The uterus is an important site for fetal development and plays an important role in life formation and development in mammals (Browne et al., 2015). At the same time, the uterus is also a functionally complex endocrine organ that can secrete many bioactive substances to regulate local and systemic physiological and pathological processes and ensure the dynamic equilibrium in interactions and endocrine secretion with other organs and tissues. During pregnancy, the uterus of the mother will undergo a series of changes as the fetus develops, including the increase in uterine volume and weight, the increase in blood flow in various parts of the uterus, and changes in nerve distribution, tissue hormone level, and their receptors. When the fetus is full term, the uterus will undergo a series of changes as delivery occurs to ensure that all changes that occurred during pregnancy will recover or be restored to pre-pregnancy status to fully prepare for the next pregnancy (Kaelin Agten et al., 2018).

The uterus and ovaries are dependent on and interact with each other in morphology and regulation of endocrine secretion during recovery of reproductive function in female animals. For example, granulosa cells in the ovaries secrete E${}_{2}$, while the corpus luteum secretes P${}_{4}$. These two hormones are transported by the UOP arteriovenous anastomosis to the uterus to regulate cyclical changes in the endometrium and myometrial contraction. Conversely, the endometrium and myometrium will synthesize and secrete prostaglandin (PG) family hormones, which are transported from the UOP blood vessels to ovary–corpus luteum tissues and participate in ovary–corpus luteum degeneration. Currently, many studies proved that a large number of miRNAs are present in ovary and uterus tissues. These miRNAs play important roles in granulosa cell proliferation, sex hormone synthesis and secretion, corpus luteum apoptosis, and endometrial regeneration and remodeling. It is reported that miR-23a, miR-23b, miR-542-3p, miR-211, and miR-17-5p can target several key genes in the PGs-P${}_{4}$-E${}_{2}$ axis in ovary–uterus tissues, such as the expression of *COX2*, *StAR*, and *CYP19A1* to participate in regulating granulosa cell proliferation, differentiation, and steroid biosynthesis and secretion (Donadeu et al., 2012). At the same time, Akhtar and Haqqi (2012) found that miR-199a can target and regulate COX2 expression to participate in the synthesis of intrauterine PGF${}_{2\alpha }$ so that the former plays a key role in uterine contraction. In contrast to previous studies, we carried out an alignment analysis of differentially expressed miRNAs in the ovary and uterus libraries and found one common miRNA, which was miR-200a. In addition, four target-prediction software programs, including TargetScan, miRDB, miRWalk, and miRTarBase, were employed, and we found 39 target genes for miR-200a. These were combined with KEGG signaling pathway enrichment analysis, which showed that a miRNA-target gene pair, miR-200a-*ZEB1*, was involved in microRNAs in cancer. At the same time, another miRNA-target gene pair, miR-200a-*YAP1*, is involved in the Hippo signaling pathway. MiR-200a is a member of the miR-200 family, and a large volume of studies have proved that it participates in many physiological regulation processes. For example, Williams (2014) showed that miR-200a can directly inhibit *STAT5b* increase and can decrease *20* α *-HSD* expression to regulate myometrial P${}_{4}$ level to participate in regulating uterine contraction. In fact, it is worth mentioning that miR-200 family members can regulate the expression of ZEB family members (*ZEB1* and *ZEB2*) to inhibit genes associated with smooth muscle contraction (*CX43* and *OTR*) to maintain myometrial quiescence. Conversely, when both *ZEB1* and *ZEB2* simultaneously decrease, *OTR* and *CX43* genes will be significantly upregulated and induce myometrial excitation. At the same time, a study found that elevation in the concentration of P${}_{4}$ secreted by ovaries can induce *ZEB1* expression, thereby weakening the inhibitory effects of miR-200 family members on ZEB family members (Wu and DeMayo, 2017). Similarly, Wu and DeMayo (2017) conducted a study on in vitro transfected uterine trophoblast cells and found that miR-200a inhibition can increase the proliferation, migration, and invasion of uterine trophoblasts, while transfection with *ZEB1* siRNA can inhibit miR-200a expression. Therefore, the aforementioned studies showed that miR-200a may target ZEB family members (such as *ZEB1*) in the postpartum mother to participate in postpartum uterine remodeling, including uterine involution and repair of the intrauterine environment. In addition, we also noticed that another target gene of miR-200a is *YAP1*, which is a core gene in the Hippo pathway. This pathway plays a role in regulating in vivo balance, oncogenesis, and regeneration. Chen et al. (2011) reported that the YAP1/Hippo pathway plays a crucial role in controlling the differentiation of myofibroblasts and fibrosis development. At the same time, Song et al. (2016) found that compared with normal endometrium, *YAP* overexpression in the YAP/Hippo signaling pathway will not only lead to endometriosis but also upregulate *CTGF* and *BCL-2* expression to increase proliferation and decrease apoptosis. In summary, many studies showed that some regulatory circuits may be present in ovaries and uterus in the postpartum mothers. That is, the *ZEB1*-miR-200a-*YAP1* pathway, which connects the uterus–ovary axis and PG-E${}_{2}$/P${}_{4}$ endocrine regulatory networks to participate in the postpartum maternal uterine remodeling, plays important regulatory roles during this process.

In conclusion, we constructed postpartum ovine ovary and uterus libraries and successfully screened out eight known differentially expressed miRNAs, of which one differentially expressed intersection miRNA-200a. In addition, bioinformatics analysis found two pairs of miRNA-target genes – miRNA-200a-*ZEB1* and *YAP1* – may participate in regulating postpartum maternal uterus morphology and function recovery through the ovary–uterus exits constructed by *ZEB1*-miR-200a-*YAP1* pathway. This provides a direction and a solid foundation for an in-depth examination of the molecular network regulatory mechanisms in postpartum uterine involution in female animals.

## Supplement

10.5194/aab-64-167-2021-supplement The supplement related to this article is available online at: https://doi.org/10.5194/aab-64-167-2021-supplement.

## Data Availability

The data are available from the corresponding author upon request.

## Code availability

SPSS software (version 20.0) is available at (IBM, 2021).
