# Supplementary material for: Comparative analysis of differentially expressed miRNAs related to uterine involution in the ovine ovary and uterus
Source: Arch Anim Breed. 2021 May 12;64(1):167–75. doi: 10.5194/aab-64-167-2021 (PMC8161056; doi:10.5194/aab-64-167-2021)
Supplement: The supplement related to this article is available online at: https://doi.org/10.5194/aab-64-167-2021-supplement. [file aab-64-167-supplement.pdf]

Supplement of Arch. Anim. Breed., 64, 167–175, 2021
https://doi.org/10.5194/aab-64-167-2021-supplement
© Author(s) 2021. CC BY 4.0 License.

Open Access
Archives Animal Breeding

Supplement of

Comparative analysis of differentially expressed miRNAs related to uterine involution in the ovine ovary and uterus

Heng Yang et al.

Correspondence to: Heng Yang (yh20183007@swu.edu.cn) and Gaofu Wang (29414467@qq.com)

The copyright of individual parts of the supplement might differ from the article licence.

Table S1A the primer sequence of miRNA (ovary)

	miR_name	Primer
UFO/USO	oar-miR-362	GCAATCCTTGGAACCTAGGTGTGAGT
	oar-miR-200a	GGGGTAACACTGTCTGGTAACGATGTT
	oar-miR-200c	GGTAATACTGCCGGGTAATGATGGA
	oar-miR-200b	GGGGTAATACTGCCTGGTAATGATG
	oar-novel-miR-719-5p	CGGCGGGGGCGGTCC
	oar-novel-miR-296-5p	GTCCTTCATTCCACCGGAGTCTG
	U6	CAAGGATGACACGCAAATTCG

Table S1B the primer sequence of miRNA (uterus)

	miR_name	Primer
UFU/USU	oar-miR-200a	GGGGTAACACTGTCTGGTAACGATGTT
	oar-miR-99a	CCGAACCCGTAGATCCGATCTTG
	oar-miR-133	TCCGTTTTGGTCCCCTTCAAC
	oar-miR-379-5p	CGTGGTAGACTATGGAACGTAGGC
	oar-novel-miR-1185-3p	CCCGGCCTGGAATGTAAAGAAGTATGTAT
	oar-novel-miR-1109-5p	GGCCCGTCCCGTGCGTCAA
	U6	CAAGGATGACACGCAAATTCG

Table S1C the primer sequence of target genes

	Gene	Primer (5'–3')	Product size (bp)
UF/US	ZEB1	F: TGGTGCTGGTACTGATGCTGAT	287
		R: CCCACTGGTCTTTACCCAATAGA	
	YAP1	F: GACAGCGGACTGAGCATGAG	108
		R: CAGGGTGCTTTGGTTGATAGTG	
	β-catenin	F: GGGAGTCCGCATGGAAGAAA	122
		R: GCAGCTGCACAAACAATGGA	
